# Queen Victoria's Jubilee Institute for Nurses

**Published:** 1909-06-19

**Authors:** D. F. Pennant

**Affiliations:** Honorary Secretary


### QUEEN VICTORIA'S JUBILEE INSTITUTE FOR NURSES.

By D. F. PENNANT, Honorary Secretary.

My attention has been drawn, I am afraid rather late in the day, to the article on "Nursing Administration" in The Hospital of May 15. The Council of the Queen's Institute always welcomes criticisms of its work, particularly when they proceed from a periodical of the high standing of The Hospital, and is always grateful for any assistance in bringing before the public notice the essential difficulties which exist in successfully promoting the nursing of the sick poor in their own homes. But as the article in question, in some respects, seems calculated to create a wrong impression of the actual condition of the Institute's work, perhaps you will allow me shortly to explain exactly how matters stand.

Under the Charter incorporating the Institute, there is committed to it "the promotion and provision of improved means for nursing the sick poor." With so vast a field of work it must always be obvious that at any given time the Institute can only select the more urgent or more important methods of carrying out its mission which present themselves, and that it must always leave aside much useful work which would conduce to the improvement of the nursing of the sick poor. In this sense, but in this sense only, is the Institute always "in difficulties," unable, that is, to carry out all the work which it would wish. In any other sense, the record of the year 1908 is a record of difficulties overcome, and there is, in truth, no ground whatever for depression in connection with the Institute's work. More money indeed is always needed, for district nursing is spreading fast, and the demands made on the Institute for advice and assistance grow even faster. But the work accomplished is scarcely done justice to in your article. It is fair to say that it has been difficult to give the figures as to the nurses so as to make them clear to those who are not already familiar with the method of training Queen's Nurses.

The facts are these:

Every Queen's nurse, in addition to three years' hospital training, is given at least six months, not a year's, training in district work—in 1908 there were 174 so trained at the expense of the Institute and its Scottish branch in large homes; the seven who for special reasons received hospital training, and the thirty who received training in midwifery, must also have been trained in district work before becoming Queen's nurses. In addition to these, 67 received the necessary district training to qualify them as Queen's nurses from homes of sufficient size to enable them to train, for use on their own staffs, making in all 241 added to the Roll of Queen's nurses during the year. The year 1909 should show an increase in the number of nurses trained both at the expense of the Institute and by homes for their own staff.

There is now no shortage of vacancies in the training centres, and the number of nurses in training is being increased as fast as the supply of hospital trained nurses will allow.

The requirements of the large training homes necessitate a constant succession of nurses being sent to them for training throughout the year, and this makes it more difficult to secure the number of nurses required, than would be the case if they all commenced training at stated times.

Again, with few exceptions, the affiliated nursing homes in the provinces which employ a Superintendent and not less than five nurses, are now training as Queen's Nurses all the nurses they require for their own use. The number of nurses, then, wanted to train as Queen's Nurses is rapidly increasing. Miss Nightingale has said that it is the best nurses who are wanted for work among the poor. Who will help us to get more to come forward?

It is, of course, true that the necessity for training so many nurses is due to the large number of resignations, but the writer of the article has not quite appreciated the figures. There were in 1908, 203 resignations. But there were 241, not 211 nurses trained, and to these, to make a fair comparison, must be added 33 who rejoined, having resigned in previous years, a total of 274, or 71 more than the resignations.

Then, of the nurses resigned, in the case of a certain number, the resignations are only temporary, due to breakdown in health; securing of a post under an Association which ultimately affiliates to the Institute, and other like causes. But the great loss of nurses is due to marriage. Marriage ends the nurse's career for the time being.

One word as to the cost of inspection. It is only Queen's Nurses who are inspected by the Institute's inspectors, and of these only those in the country districts are midwives. There is then no room for a combination of the office of inspector of midwives under a County Council with that of the Institute's inspector of Queen's Nurses, who covers an area commensurate with several counties. It is the Superintendents of the County Nursing Associations who naturally combine their functions with those of official Inspector of Midwives, and the Institute has always urged that wherever possible, this should be arranged.

The Prince of Wales, on the occasion of his visit to Midsomer Norton, Somerset, on June 23, will present the Honorary Associate's badge of the Order of St. John of Jerusalem in England, of which he is Grand Prior, to Dr. Worger, of Radstock, and the Society's vellum certificate to Mr. Lloyd Harvey, of Radstock, for services rendered by them for ten years to the cause of first aid to the injured.

